# Mediterranean diet affects the metabolic outcome of metabolic dysfunction-associated fatty liver disease

**DOI:** 10.3389/fnut.2023.1225946

**Published:** 2023-10-10

**Authors:** Yuji Xiao, Xue Zhang, Dongxin Yi, Fangyi Qiu, Lei Wu, Yiyong Tang, Ningning Wang

**Affiliations:** ^1^School of Public Health, Dalian Medical University, Dalian, Liaoning, China; ^2^Bishan Hospital of Chongqing Medical University, Chongqing, China; ^3^The Second People’s Hospital of Dalian, Dalian, Liaoning, China; ^4^Department of Radiobiology, Beijing Key Laboratory for Radiobiology, Beijing Institute of Radiation Medicine, Beijing, China; ^5^Department of Cardiovascular Ultrasound, The Second Affiliated Hospital of Dalian Medical University, Liaoning, China; ^6^Department of Nutrition and Food Hygiene, School of Public Health, Dalian Medical University, Dalian, Liaoning, China; ^7^Global Health Research Center, Dalian Medical University, Dalian, Liaoning, China

**Keywords:** mediterranean diet, metabolic dysfunction-associated fatty liver disease (MAFLD), insulin resistance, intrahepatic lipids, hepatic steatosis, inflammation

## Abstract

The prevalence of metabolic dysfunction-associated fatty liver disease (MAFLD) is on the rise globally. It is currently one of the most prevalent liver diseases and one of the world’s important public health problems. At present, there is no consensus on a pharmacological treatment for MAFLD. By contrast, lifestyle interventions based on exercise and a balanced diet are considered to be the cornerstone of MAFLD management. Mediterranean diet (MD) have a large content of polyphenols, polyunsaturated fatty acids, oleic acid, carotenoids and fiber, which carry out antioxidant, anti-inflammatory and antibacterial benefits. It has been considered to reduce the incidence rate of cardiovascular disease and type 2 diabetes. The purpose of this narrative review is therefore to summarize and analyze the evidence for the effect of MD on metabolic outcomes in MAFLD patients.

## 1. Introduction

Metabolic dysfunction-associated fatty liver disease (MAFLD) refers to a clinico-pathologic syndrome characterized by an excessive deposition of fat in hepatocytes, unrelated to consumption of alcohol and other overt liver-damaging factors. The total worldwide prevalence of MAFLD diagnosed by imaging in 2015 is estimated to be 25.24% (1). Recently, a trend analysis showed that the prevalence of MAFLD increased from 25.3% [(1990–2006) to 38.2% (2016–2019), (2)]. Considering the increase incidence of obesity, diabetes and other factors, it is expected that the prevalence of MAFLD and the proportion of patients with advanced liver disease will continue to increase. In 2020, international experts unanimously recommended that nonalcoholic fatty liver disease (NAFLD) be renamed MAFLD and that the criteria for a MAFLD positive diagnosis be based on histology (biopsy), as evidenced by the accumulation of liver fat (liver steatosis) by imaging or as a blood biomarker, and any of the following 3 criteria, namely overweight/obesity, evidence of type 2 diabetes mellitus (T2DM), or metabolic disorders (2, 3). Patients with metabolic steatosis of the liver with overt steatohepatitis and progressive fibrosis have a significantly increased risk of adverse outcomes, including overall rates of mortality, liver complications and mortality. Kim et al. (4) analyzed the data from 7,761 participants in the third national health and nutrition review survey conducted in 2015 and their associated mortality, found that during the median follow-up period of 23 years, patients with MAFLD had a 17% greater risk of death from all causes (risk ratio [HR] 1.17; 95% CI 1.04–1.32). Moreover, MAFLD is associated with a greater risk of cardiovascular death (4).

Obesity and diet patterns are considered to be the key factors in MAFLD development, with most MAFLD patients being overweight or obese. High-fat and high-calorie diet, sedentary lifestyle, insulin resistance (IR), metabolic syndrome (Mets) and its components (obesity, hypertension, dyslipidemia and pathoglycemia) are prevalent risk factors for MAFLD, of which T2DM is an important risk factor for MAFLD independent of obesity.

Currently, there is no consensus regarding pharmacological treatment for MAFLD. In contrast, exercise-based lifestyle interventions with a balanced diet are thought to be the cornerstone of treatment strategies for MAFLD. Of note, promoting adherence to the Mediterranean diet (MD) pattern and physical activity (PA—150 min of moderate intensity exercise per week) are more effective than any single drug choice (5, 6).

Although MD profiles may vary between countries and regions due to differences in culture, ethnicity, religion and agriculture (7), they share common features including: (1) A low saturated fat intake in butter, whole milk and red meat; (2) Monounsaturated fatty acids (MUFA) consumption is mainly contained in olive oil; (3) Adequate balance of polyunsaturated fatty acids (omega-6 and omega-3) is mainly accounted for the consumption of fish, shellfish and nuts; (4) Low protein intakes of terrestrial animals, particularly of red meat; (5) High intake of antioxidants, present in fruits, vegetables, wine, virgin olive oil, spices and herbs, and (6) High fiber intake from plant-based sources, such as vegetables, fruits, whole grains, legumes and nuts (7, 8). In order to ensure the reliable usage of the diet regime, the dietary structure and specific dosage recommended by the Mediterranean Diet Foundation (2011) include (1) every meal: olive oil and vegetables ≥2 serves, fruits 1–2 serves, breads and cereals 1–2 serves; (2) daily: nuts 1–2 serves, dairy foods 2 serves; (3) weekly: legumes ≥2 serves, fish and seafood ≥2 serves, eggs 2–4 serves, poultry 2 serves, red meat and sweets <2 serves; (4) red wine: in moderation and respecting social beliefs (7, 9). Based on current available intervention studies, following MD of 50–60% carbohydrates, 15–20% proteins, and less than 30% fat can effectively improve liver metabolic indicators in patients with MAFLD, achieve intervention effects, and reduce the occurrence and development of MAFLD (10–12). The protective effect of MD has been considered in most studies and may be primarily due to fits anti-inflammatory and antioxidant properties (13, 14). For example, the ability of MD to reduce the risk of MAFLD onset and progression may be attributed to the nutritional and health effects of bioactive compounds and phytochemicals with antioxidant and anti-inflammatory capacities, such as fibre, MUFA, omega-3 fatty acids and phytosterols (14, 15). Emerging evidence continues to demonstrate that increasing MD adherence in MAFLD patients can improve levels of intrahepatic lipids (IHL), IR and other markers of metabolic risks (11, 12, 16).

For this, we searched PubMed, Web Of Science and Google scholar from 1995 to 2023. The terms we used for retrieving articles were Mediterranean diet, MD, metabolic dysfunction-associated fatty liver disease, MAFLD, non-alcoholic fatty liver disease, NAFLD, insulin resistance, intrahepatic lipids, hepatic steatosis and inflammation, with the strategies as follows ((Mediterranean diet[Title/Abstract]) OR (MD[Title/Abstract])) AND ((((metabolic dysfunction-associated fatty liver disease[Title/Abstract]) OR (MAFLD[Title/Abstract])) OR (nonalcoholic fatty liver disease[Title/Abstract])) OR (NAFLD[Title/Abstract])) OR ((((insulin resistance) OR (intrahepatic lipids) OR (hepatic steatosis) OR (inflammation)).

## 2. The effect of MD on IR in MAFLD

IR is defined as a biological response to changes in circulating insulin, which is the pathophysiological basis of obesity and T2DM, and is also related to a variety of pathological conditions such as MAFLD, cognitive impairment, endothelial dysfunction, chronic kidney disease, polycystic ovary syndrome and certain endocrine tumours including breast cancer (20). As the first hit of MAFLD, IR is closely related to the initiation of MAFLD. Under the condition of IR, adipose tissue develops resistance to the anti-lipolysis of insulin and the release of fatty acids increases (21), and in turn, elevated insulin level promote the synthesis of liver triglycerides in the case of increased lipolysis and/or increased fat intake (22). Compared with other nutritional interventions, MD was significantly associated with IR improvement in obese individuals (20). Similarly, in a single-arm study involving 46 overweight or obese subjects, Gelli et al. (12) found that glucose concentration and homeostasis model assessment of insulin resistance (HOMA-IR) were significantly improved after 6 months of independent MD intervention in all patients (see Table 1) (12). As part of a healthy lifestyle, a diet that reduces energy always contributes to weight loss and IR reduction in these patients (23). International guidelines suggest that the first step in the treatment of MAFLD is to limit the intake of calories, fats (saturated fatty acids, trans-fatty acids) and fructose, while increasing the intake of fiber and polyunsaturated fatty acids (24), and MD fits neatly into this dietary structure. In a meta-analysis of Kawaguchi et al. (16), MD was suggested to significantly reduce HOMA-IR compared with the control diet (SMD: –0.34; 95% CI: −0.65 to −0.03; *p* = 0.03) (see Table 1) (16). Typically, MD compliance was assessed using the 14-point Screening Questionnaire Mediterranean Diet Adherence Screener (MEDAS), with higher scores indicating higher adherence (17, 25). In a 12-week randomized controlled study, George et al. (17) compared the effect of MD and low-fat diet (LFD) on the IR index (HOMA-IR) in MAFLD patients, showing a reduction in HOMA-IR of 0.5 unit from baseline in the MD group and 1 unit reduction in the LFD group. Notably, compliance with MD increased by 2.7 units (6.5 ± 2.0 to 9.2 ± 1.9, with a maximum possible score of 14, *p* < 0.005), far exceeding compliance in the LFD group (5.4 ± 2.0 to 6.4 ± 2.3, with the highest possible score of 9, *p* = 0.035) (see Table 1) (17). It can be predicted that MD is more friendly to MAFLD patients and more likely to be adopted in the long term. Energy restriction and PA are recognized to be effective for MAFLD, but some diets involving excessive and/or rapid weight loss, such as high fat diet and extremely low carbohydrate diet, may actually lead to or accelerate disease progression and induce IR (26). In a study that included 584 consecutive outpatients with one or more CVD risk factors, such as T2DM, arterial hypertension, obesity/overweight and dyslipidemia, who were divided into low, intermediate, and high MD adherence, Baratta et al. (11) found that good compliance to MD diet is related to lower IR in patients of MAFLD and an inverse relationship between MD compliance and MAFLD prevalence (see Table 1) (11). Therefore, it is noteworthy that MD intake is inversely dependent on the severity of MAFLD in suffered patients.

**Table 1:** Effect of MD on metabolic outcomes of MAFLD patients.

Metabolic Outcome	Number of participants	Study Design	Results	Author
Insulin resistance	250 NAFLD participants selected for a meta-analysis	A meta-analysis included six randomized controlled trials	MD improved IR in patients with MAFLD.	Kawaguchi et al. (16)
	584 overweight/obese adult patients with ≥1 risk factor for CVD events	A randomized controlled trial	Good compliance to MD was associated with lower IR.	Baratta et al. (11)
	42 participants randomised to LFD and MD with 12 weeks intervention	A parallel multicentre randomized controlled trial	Both MD and LFD improve IR.	Georg et al. (17)
	46 adults with MAFLD received 6-mo of clinical and a dietary intervention (based on MD)	An observational study	HOMA-IR values significantly improved.	Gelli et al. (12)
Intrahepatic lipid	46 adults with MAFLD received 6-mo of clinical and a dietary intervention (based on MD)	An observational study	Both MD and LFD improve IHL.	Gelli et al. (12)
	42 participants randomised to LFD and MD with 12 weeks intervention	A parallel multicentre randomized controlled trial	Both MD and LFD moderate improve IHL.	Georg et al. (17)
	36 female adults (MAFLD: control = 19:17) received surveys and medical examinations	A randomized controlled trial	Total energy intake was positively associated with both HFF and IHL.	Cheng et al. (18)
Hepatic steatosis	250 NAFLD participants selected for a meta-analysis	A meta-analysis included six randomized controlled trials	MD improved hepatic steatosis in patients with MAFLD.	Kawaguchi et al. (16)
	46 adults with MAFLD received 6-mo of clinical and a dietary intervention (based on MD).	An observational study	The percentage of patients with a grade of steatosis equal to or higher than 2 was reduced from 93 to 48% and steatosis regressed in 9 patients (20%).	Gelli et al. (12)
	12 non-diabetic subjects with biopsy-proven MAFLD	A randomized controlled trial	Patients experienced a 38% reduction in liver steatosis following MD intervention after 6 wk. of treatment.	Ryanet et al. (10)
Inflammation	180 adults with Mets received a 2 years randomized, single-blind trial (based on MD)	A randomized controlled trial	After 2 years of treatment, the intervention group had more decreased inflammatory markers (CRP, IL-6, IL-7, and IL-18).	Esposito et al. (19)
	46 adults with MAFLD received 6-mo of clinical and a dietary intervention (based on MD)	An observational study	Systemic inflammation indexes [platelet to lymphocyte ratio (PLR) and neutrophil to lymphocyte ratio (NLR)] were significantly reduced 6 months after intervention.	Gelli et al. (12)

## 3. The effect of MD on IHL deposition

The global obesity pandemic has paved the way for the dramatic increase in MAFLD. Intrahepatic lipid (IHL) accumulation is most commonly observed in cases of obesity, and together with IR as the first hit of MAFLD. Briefly, lipids delivered to the liver exceed the sum of hepatic lipid oxidation and secretion, IHL content begins to rise, and nonalcoholic fatty liver can develop (27). Further, toxicity caused by IHL accumulation (lipotoxicity) may also drive further steps in this disease such as inflammation, liver injury and IR. In a set of randomized controlled trials, George et al. (17) assessed the effect of 12-weeks of MD and LFD intervention on IHL in subjects with MAFLD using proton magnetic resonance spectroscopy (1H-MRS) as the non-invasive gold standard method for measuring IHL, with moderate improvement in IHL in both dietary intervention groups (compared with baseline, IHL in MD group decreased by 8%, and that in LFD group decreased by 17%) (see Table 1) (17). In addition, Cheng et al. (18) used 1H-MRS and dual echo MRI to evaluate the relationship between dietary nutrient intake and hepatic lipid content in female patients with MAFLD. Results showed that energy, protein, saturated fatty acid intake was higher in patients with MAFLD, and total energy intake was positively related to liver fat fraction (HFF) and IHL, underscoring the critical role of dietary factors in the accumulation of IHL and the development of MAFLD (see Table 1) (18). However, the evidence in this regard is relatively limited, and further research is warrant to illustrate the mechanism of MD intervention in reducing IHL in MAFLD patients.

## 4. The effect of MD on hepatic steatosis in MAFLD

Hepatic steatosis is considered to be the first step in the pathophysiological continuum of MAFLD, with numerous consequences ranging from progression to chronic liver disease and associated mortality, to deterioration of IR and T2DM, thus becoming an independent factor of CVD (28). The steatotic liver will further contribute to the development of IR as a result of impaired insulin clearance from portal blood (29). In a meta-analysis of 6 randomized controlled trials led by Kawaguchi et al. (16) revealed that MD significantly reduced the fatty liver index (FLI) (SMD: –1.06; 95%CI: −1.95 to −0.17; *p* = 0.02), demonstrating that MD improved hepatic steatosis in patients with MAFLD disease (see Table 1) (16). Gelli et al. (12) performed a 6-month MD-based dietary intervention in 46 adults with MAFLD, and uncovered that the proportion of patients with hepatic steatosis grade above or equal to 2 decreased from 93 to 48%, and 9 patients had a 20% regression of steatosis (see Table 1) (12). The degree of hepatic liposteatosis under ultrasonic imaging was graded as absent (0), mild (1, the presence of bright echo or elevated hepatic contrast), moderate (2, the presence of bright echo and elevated liver and renal contrast, and vascular blurring) and severe (3, severe steatosis with evidence of posterior bundle attenuation and invisible diaphragm) (30). In a randomized crossover intervention trial where 12 non-diabetic MAFLD patients were recruited for dietary intervention, Ryan et al. (10) compared MD with a low-fat, high carbohydrate diet. After 6 weeks of treatment, patients assigned to MD intervention had a 38% reduction in hepatic steatosis (as assessed by 1H-MRS) compared with a low-fat, high-carbohydrate diet, regardless of weight loss or change in waist circumference (see Table 1) (10). Reversing hepatic steatosis or controlling its progression is essential for the prevention and treatment of MAFLD, and MD can be applied as a beneficial pharmaconutritional therapy in this pivotal reversible course of MAFLD.

## 5. The effect of MD on inflammation in MAFLD

The advantage of MD lies in the balanced combination of various beneficial foods or nutrients. Although it is still not possible to fully distinguish which foods or nutrients in MD are responsible for the main anti-inflammatory effect, cumulative evidence suggests that multiple nutrients from a range of different foods (not just a few specific foods) have a synergistic and interactive role in the attenuation of inflammation. Evidence from several epidemiologic studies supports this hypothesis, suggesting that individuals consuming a high-quality diet have a lower inflammatory state, independent of metabolic risk factors (31, 32). In a randomized controlled trial over 2-years, 180 subjects with Mets were assigned to MD (received detailed advice on how to increase daily intake of whole grains, fruits, vegetables, nuts, and olive oil) or control prudent diet (carbohydrates, 50–60%; proteins, 15–20%; total fat, < 30%), inflammatory markers (CRP, IL-6, IL-7, and IL-18) were significantly decreased after 2 years of MD diet intervention (see Table 1) (19). It is widely accepted that MD is rich in omega-3 fatty acids, and its intake has been inversely correlated with circulating inflammatory markers and triglyceride concentrations. Omega-3 fatty acids appear to mediate their anti-inflammatory effects through binding to the G protein-coupled receptor 120 and inhibiting the activity of the NLRP3 inflammasome (33, 34). As part of a 6-month dietary and clinical intervention (based on MD) in 46 adults with MAFLD, the indices of systemic inflammation indexes [platelet to lymphocyte ratio (PLR) and neutrophil to lymphocyte ratio (NLR)] were significantly reduced 6 months after intervention (see Table 1) (12). Thus far, it has been universally accepted that the protective effect of MD may be primarily due to the anti-inflammatory and antioxidative properties of its constituents. The well-known PREDIMED study has confirmed that MD has high polyphenol content and revealed an overall increase in antioxidant endogenous systems observed in patients assigned to supplement extra virgin olive oil (EVOO) and nuts (35). In particular, the ability of MD to reduce the risk of development and progression of MAFLD is attributed to the nutritional healthcare effects of bioactive compounds and phytochemicals with antioxidant and anti-inflammatory capabilities, such as fiber, MUFAs and omega-3 fatty acids and phytosterol (14, 15), and with EVOO being the main source of fat in MD and may be the main driving force for its positive impacts on hepatic structure and function (20). The case in point is hydroxytyrosol, a derivative of EVOO, can protect the liver from metabolic damage (e.g., lipid peroxidation, steatosis, ischemia–reperfusion injury, inflammatory response) by scavenging free radicals, enhancing antioxidant activity, and improving mitochondrial function (36–38); likewise, among non-flavonoids, resveratrol has also been shown to exert liver protective activity by maintaining body homeostasis through the interaction of blood vessels, platelets, and coagulation, as well as the fibrinolytic system of plasma (39, 40). Vitamins are also an important component of MD and vitamin C, vitamin E can be considered as dietary antioxidants, they play a crucial role in preventing the progression of MAFLD by reducing cellular stress (41). Recent evidence also points to olive oil microconstituents, MD wild greens (ie, the heart of *Cynara* and the leaves of the rest of the plants), as well as a diet rich in antioxidants (like herbal drinks and coffee), its precisely the trace components in these substances that exert anti-inflammatory and antioxidant activities associated with reduced platelet activating factor (PAF) levels or activity which is related to the FLI and liver disease, suggesting potential new mechanisms of the diet-disease associations (42–46). Based on research so far, the effects of the different dietary components may be too small to be perceptible, and their contributions are difficult to distinguish from one another in the human body, but their cumulative effects may be large enough to discern.

## 6. Discussion

**Figure 1:**
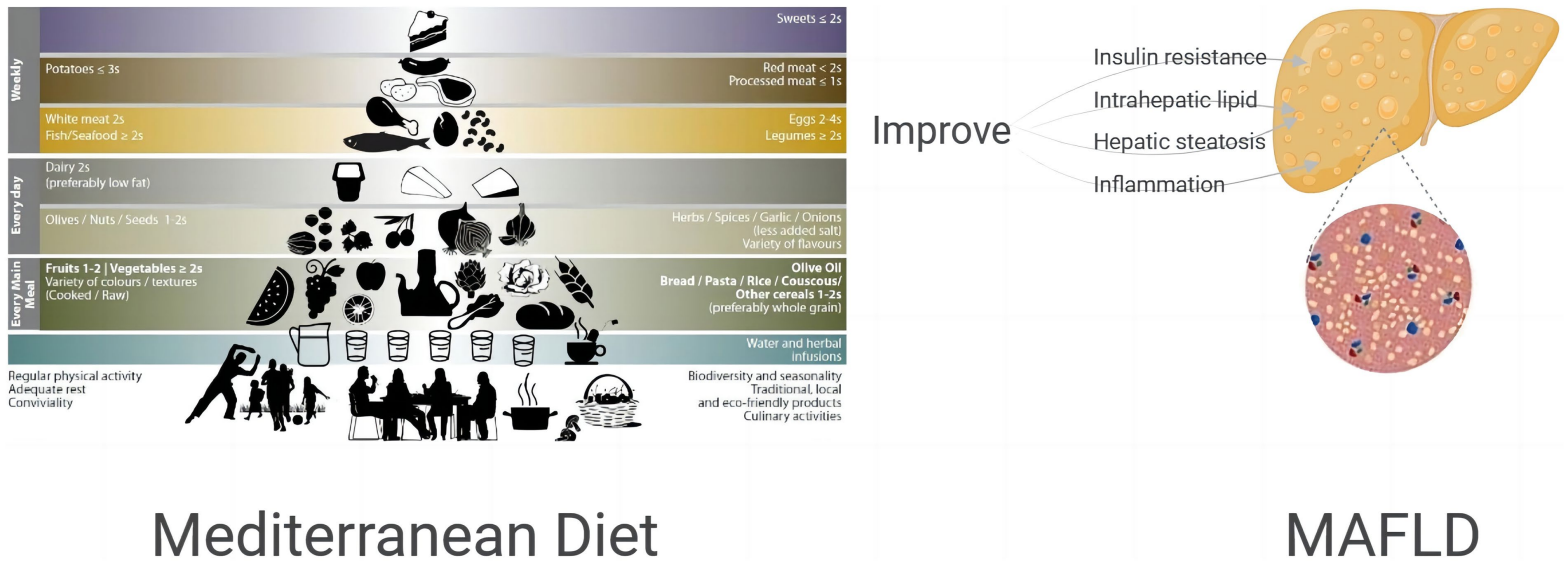
Effect of MD on insulin resistance, intrahepatic lipid, hepatic steatosis, inflammation of MAFLD patients.

In this review, twelve studies detailing the improvement of IR, IHL deposition, hepatic steatosis, hepatic inflammation and oxidative stress in MAFLD patients (see Figure 1) through MD pattern lifestyle modification were collected and analyzed, suggesting a beneficial effect of MD on metabolic outcomes in MAFLD patients. Through comprehensive collection of scattered clinical population evidence, including high quality meta-analysis, clinical randomized controlled trials, observational study and prospective studies, we have in detail explained the beneficial effects of MD on metabolic indicators, providing theoretical and practical basis for the prevention and development of MAFLD, which is the innovation and value of this review. All of the available evidence is in favor of recommendation of MD in patients with MAFLD. In addition, long-term exercise training also resulted in marked but more moderate improvements in IHL as well as hepatic metabolism, as proposed in a review by Brouwers et al. (47). In a multicenter prospective randomized trial, subjects with MAFLD and Mets experienced reductions in both IHL content and liver stiffness after 6-and 12-month of MD-based personalized nutrition intervention and PA intervention (47). Growing evidence supports that the adoption of MD dietary pattern in combination with PA intervention may be more effective in improving MAFLD.

Similarly, based on an one-year lifestyle interventions by Jenning et al. (2020), energy-restricted MD with PA and behavioral support can reduce weight and improve risk factors related to CVD (48). The reason is that factors such as PA can also alter glucose regulation through plasma branched-chain amino acids (BCAAs), playing an additional beneficial role in overweight/obese individuals (49). However, their interaction has not been studied thoroughly, further comprehensive interaction analysis is needed to investigate the potential synergies between lifestyle components.

MAFLD is associated with visceral obesity, IR, dyslipidemia and chronic inflammation, all of which are also characteristics of Mets, and MD may ameliorate MAFLD mainly through the regulation of these disorders (50). In particular, the anti-inflammatory and antioxidant effects of MD, as well as the lipid-lowering effect and metabolite production mediated by intestinal microflora are the main mechanisms affecting metabolic health and MAFLD (50). A meta-analysis of Italian and Spanish data concludes that one possible explanation for the success of MD in human health is the synergy of all the components of MD, rather than individual food or nutrient intake (19). Since MD is based on the combination of compounds, for example polyphenols, fiber, vitamins and other biomolecules, and has a high MUFA content, MD is related to the overall better metabolic status (19, 50). It has been proved that consuming a diet rich in MUFA improves the HOMA-IR and fasting proinsulin levels to prevent MAFLD development, improves blood lipid levels, reduces body fat accumulation, and decreases postprandial adiponectin expression in IR subjects (51, 52). The combination of macronutrients, micronutrients and various bioactive factors in MD appears to be a valuable dietary mode, which can not only reduce weight but also accompanies the metabolic benefits of MAFLD (50). Due to the high prevalence of MAFLD in the general population and the potentially serious risk to health outcomes, treatment should be given immediately after diagnosis.

As part of hypocaloric treatments, MD has been demonstrated to significantly improve IR in obese individuals compared with other hypocaloric diet approaches (53), even with less than 5% weight loss (54). It is worth mentioning that different weight loss goals can be set for MAFLD patients based on personalized NASH-related indicators. In fact, reducing 5 to 7% of initial weight is sufficient for patients with hepatic steatosis, and for patients with suspected or confirmed NASH, a higher weight loss target of 7 to 10% of initial weight is recommended (55). According to cross-sectional and longitudinal studies, MAFLD patients adhering to MD are less likely to develop steatohepatitis (56). Randomized controlled crossover experiments have illustrated the advantages of MD over LFD in improving insulin sensitivity, metabolic parameters and hepatic steatosis (10, 57). However, rapid and excessive weight loss should be avoided targeting MAFLD patients. In this regard, a very low– caloric diet is usually not recommended, and consuming 800 kcal or less per day carries a risk of protein and calorie malnutrition and may aggravate liver fibrosis and necrosis (26, 58).

The current review presents and discusses the limited evidence about MD for reversal of metabolic outcomes in MAFLD patients. Further studies with a particular focus on *in vivo* approaches are required to determine the therapeutic potential of MD in humans and to better understand its biological activity and mechanisms of action at different tissue levels.

## 7. Conclusion

The gathered evidence suggests that MD is beneficial for patients with MAFLD and can be used as a therapeutic diet, especially when MD is combined with behavioral support such as PA (150 min of moderate intensity exercise per week), which is more capable of further improvement in IR, IHL, hepatic steatosis, while the potential mechanism of action of specific components remains to be explored. Therefore, better adherence to the MD model of lifestyle may be profitable to the prevention and reversal of MAFLD.

## Author contributions

YX and XZ collaborated on writing the original manuscript and the figure design. DY, FQ, LW reviewed the manuscript. NW and YT designed the study and revised the manuscript. All authors contributed to the article and approved the submitted version.
